# Effects of dietary physical or nutritional factors on morphology of rumen papillae and transcriptome changes in lactating dairy cows based on three different forage-based diets

**DOI:** 10.1186/s12864-017-3726-2

**Published:** 2017-05-06

**Authors:** Bing Wang, Diming Wang, Xuehui Wu, Jie Cai, Mei Liu, Xinbei Huang, Jiusheng Wu, Jianxin Liu, Leluo Guan

**Affiliations:** 1 Institute of Dairy Science, College of Animal Sciences, Zhejiang University, Hangzhou, 310058 China; 2 MoE Key Laboratory of Molecular Animal Nutrition, Zhejiang University, Hangzhou, 310058 China; 3 Department of Veterinary Medicine, College of Animal Sciences, Zhejiang University, Hangzhou, 310058 China; 4 Department of Agricultural, Food and Nutritional Science, University of Alberta, Edmonton, AB T6G 2P5 Canada; 5 Current address: Beijing Key Laboratory for Dairy Cow Nutrition, College of Animal Science and Technology, Beijing University of Agriculture, Beijing, 102206 China

## Abstract

**Background:**

Rumen epithelial tissue plays an important role in nutrient absorption and rumen health. However, whether forage quality and particle size impact the rumen epithelial morphology is unclear. The current study was conducted to elucidate the effects of forage quality and forage particle size on rumen epithelial morphology and to identify potential underlying molecular mechanisms by analyzing the transcriptome of the rumen epithelium (RE). To achieve these objectives, 18 mid-lactation dairy cows were allocated to three groups (6 cows per group), and were fed with one of three different forage-based diets, alfalfa hay (AH), corn stover (CS), and rice straw (RS) for 14 weeks, respectively. Ruminal volatile fatty acids (VFAs) and epithelial thickness were determined, and RNA-sequencing was conducted to identify the transcriptomic changes of rumen epithelial under different forage-based diets.

**Results:**

The RS diet exhibited greater particle size but low quality, the AH diet was high nutritional value but small particle size, and CS diet was low quality and small particle size. The ruminal total VFA concentration was greater in AH compared with those in CS or RS. The width of the rumen papillae was greater in RS-fed cows than in cows fed AH or CS. In total, 31, 40, and 28 differentially expressed (DE, fold change > 2, FDR < 0.05) genes were identified via pair-wise comparisons including AH vs. CS, AH vs. RS, and RS vs. CS, respectively. Functional classification analysis of DE genes revealed dynamic changes in ion binding (such as *DSG1*) between AH and CS, proliferation and apoptotic processes (such as *BAG3*, *HLA-DQA1*, and *UGT2B17*) and complement activation (such as *C7*) between AH or RS and CS. The expression of *HLA-DQA1* was down-regulated in RS compared with AH and CS, and the expression of *UGT2B17* was down-regulated in RS compared with CS, with positive (*R* = 0.94) and negative (*R* = -0.96) correlation with the width of rumen epithelial papillae (*P* < 0.05), respectively.

**Conclusion:**

Our results suggest that both nutrients (VFAs) and particle sizes can alter expression of genes involved in cell proliferation/apoptosis process and complement complex. Our results suggest that particle size may be more important in regulating rumen epithelial morphology when animals are fed with low-quality forage diets and the identified DE genes may affect the RE nutrient absorption or morphology of RE. Our findings provide insights into the effects of the dietary particle size in the future management of dairy cow feeding, that when cows were fed with low-quality forage (such as rice straw), smaller particle size may be beneficial for nutrients absorption and milk production.

**Electronic supplementary material:**

The online version of this article (doi:10.1186/s12864-017-3726-2) contains supplementary material, which is available to authorized users.

## Background

Rumen epithelium (RE) consists of leaflike papillae, which not only serves as absorptive structures, but also epithelial barrier to prevent the invasion of rumen microbes and/or toxins [1]. The morphology of the rumen epithelium plays a critical role in coping with altered dietary regimens in ruminants [2]. During the dietary transition period, the morphology of RE could be changed by diets with different energy density, which may be attributed to the varied volatile fatty acids (VFAs) concentrations [3]. In addition, physical features of diet such as the finely ground compared with unground feed were also known to alter the RE morphology in dairy cows [4].

Recent studies have found that butyrate acts as a mitogenic factor and signaling molecule during cell proliferation in both cattle and humans [5–7]. The increase in the total thickness of the RE in goats after ruminal butyrate infusion has been reported, and the expression of *cyclin D1* gene was found to be associated with the increased papillae growth at the same time [8]. In addition, the changes in epithelial thickness can be linked to various cellular functions, such as cell proliferation [5] and epithelial differentiation and proliferation [9, 10]. At the transcriptional level [3, 8], it has been identified that RE morphology was related to gene targeting functions such as cellular development [11], epithelial proliferation [10], papilla size and surface area [12], and tight junctions [13]. These studies revealed aspects of the potential mechanisms by which RE morphology is regulated, but the systematic mechanisms involved in regulating rumen epithelial morphology remain to be clarified.

RNA sequencing (RNA-seq), a high-throughput sequencing based transcriptome profiling, has been proven to provide extensive quantitative and qualitative information on the expression of genes in both prokaryotes and eukaryotes [14, 15] and their potential changes under different conditions. This technique has been successfully applied to identify potential transcriptional mechanisms underlying phenotypic and physiological changes in bovine species [7, 16], leading to the findings of “potential gene markers” [17]. Therefore, in the current study RNA-seq based transcriptomic profiling was used to investigate the effects of dietary forage sources with different nutritional values (energy density) and physical forms (particle sizes) on the RE morphology and the underlying mechanism in dairy cows.

## Methods

### Animals, management, and nutritional and physical characteristics of the diets

The procedures of this study were approved by the Animal Care and Use Committee of Zhejiang University (Hangzhou, China) and were in accordance with the university’s guidelines for animal research.

A total of 18 multiparous Holstein dairy cows (6 cows per group; milk yield = 29.9 ± 2.83 kg/d, day in milk = 167 ± 25.7, parity = 3.5 ± 1.77; mean ± SD) were selected in this study. A detailed description of the experimental design and treatments has been reported previously [18]. Briefly, the 3 diets contained an identical concentrate mixture (55%, dry matter basis) and 15% corn silage, with the remaining 30% consisting of the following forage sources (dry matter basis): (1) 23% alfalfa hay and 7% Chinese wild rye hay (AH); (2) 30% corn stover (CS); (3) and 30% rice straw (RS). The crude protein content of the 3 diets was similar, but the NE_L_ values of AH, CS, and RS were 1.57, 1.45, and 1.43 Mcal/kg, respectively (Table 1). The particle size distributions of the 3 diets were evaluated using a Penn State Particle Separator according to a previous report [19]. Samples of each fraction were dried in a forced-air oven at 65 °C for 48 h and were then ground to a size of 1 mm before analysis of the dry matter (105 °C for 5 h) and natural detergent fiber (NDF) contents [20]. Dietary physical effectiveness factors and physically effective NDF (peNDF) were also calculated as described previously [19].

**Table 1:** Primers used for quantitative real-time PCR

Gene	Gene Bank ID	Primers	Primer sequence (5’-3’)	Amplicon Size	References
*HLA-DQA1*	BT020994.1	Forward	CCTTGTGGGTATCGTGGTGG	150 bp	This study
		Reverse	CGTCTAGCACGTCCACTCTT		
*PI3*	XM_005214890.3	Forward	TGACTGGGCAAGGGGAGCCG	112bp	This study
		Reverse	GGGGCCACCCCAAAGAAGCC		
*HSPB8*	BT020640.1	Forward	CAGTCTTGGCCCTTCCTTGT	176bp	This study
		Reverse	TCAATTGCGCCATCTTGCAG		
*PRSS53*	XM_015469379.1	Forward	TGCACAGCTAACATGAGCCA	148 bp	This study
		Reverse	CCAGAGCCCTTTGCACTGTA		
*DSG1*	NM_174045.1	Forward	ATCCAACTGACTTGCTCGCT	278 bp	This study
		Reverse	ACCACCACCAGTTGTGTAGC		
*BAG3*	BC133574.1	Forward	ACGCAGTAACTTGGGTGGAG	148 bp	This study
		Reverse	ACAGAGACGGCTCCAAAACA		
*CYR61*	NM_001034340.2	Forward	GATGCAACTACAACTGCCCG	116 bp	This study
		Reverse	CCACCCTTATGCTGGAAGCC		
*IGFBP3*	NM_174556.1	Forward	TTAATGCCTGCACATCCCGA	259 bp	This study
		Reverse	CCCTATGGGGCTTCAGCAAA		
*GAPDH*	BC102589	Forward	TGGAAAGGCCATCACCATCT	60 bp	[31]
		Reverse	CCCACTTGA TGTTGGCAG		
*β-actin*	NM-173979	Forward	GCCATGAAGCTGAAGATGAC	229 bp	[29]
		Reverse	CCTTCTGCAGCTCAGATATG		

### Rumen fermentation characteristics

Approximately 150 mL of rumen fluid was collected 3 h after morning feeding using an oral stomach tube [21] on day 6 of weeks 3, 6, 9, and 12, respectively. The pH was immediately determined using a portable pH meter (Starter 300; Ohaus Instruments Co. Ltd., Shanghai, China). The VFA concentration was determined via gas chromatography (GC-8A; Shimadzu Corp., Kyoto, Japan) [22]. The ammonia-nitrogen concentration was determined through steam distillation into boric acid and titration with dilute hydrochloric acid (10 mL).

### Characteristics of ruminal papillae

**Fig. 1:**
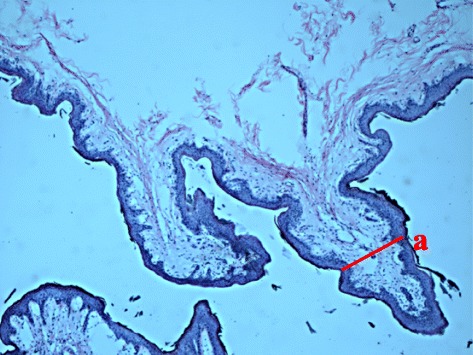
Histomicrograph showing the width of ruminal papilla (a)

The animals were slaughtered before morning feeding (7 a.m.) after 14 weeks of the feeding trial. The rumen was exteriorized and separated from the omasum after slaughter, following a previously described method [23]. A detailed description of the collected rumen papillae and histomorphometric analyses was performed according to a published procedure [8]. Briefly, the ventral sac of the rumen was chosen, as it has been determined to be the site with the highest capillary blood flow per unit weight mucosa of any location within the rumen [24]. Sterile surgical scissors were used to clip approximately 800 mg of ruminal tissue, which was then quickly washed 20 times in ice-cold PBS buffer (pH = 7.2). For histomorphometric analyses, the RE samples (approximately 2 cm^2^) including atrium ruminis, ventral rumen sac, and ventral blind sac were selected and were fixed in 4% paraformaldehyde overnight. Then, they were dehydrated, cleared, and embedded in paraffin. Sections of 8 μm in width were cut and stained using the standard hematoxylin and eosin (H&E) procedure. Papillae with almost identical shapes and sizes were embedded for paraffin sectioning and microscopic observation for histomorphological analysis. The morphological characteristics of the ruminal papillae were selected from 4 paraffin sections showing the best orientation of papillae in the median sagittal plane for evaluation using Image-Pro Plus 6.0 (Media Cybernetics Inc., Bethesda, MD). The width of rumen papillae (WP) was measured following previously described methods [8], which can be seen in Fig. 1. In the meantime, the rumen papillae were separated from the stratum basale of the RE and were snap frozen using liquid nitrogen, transferred to the laboratory, and stored at -80°C for downstream transcriptomic analysis.

### Total RNA extraction and mRNA library construction

Total RNA from the RE tissue (approximately 100 mg) was extracted with TRIZOL reagent (Invitrogen, Carlsbad, CA) according to the manufacturer’s procedures. The concentration and purity of the total RNA were measured using a NanoDrop® ND-1000 Spectrophotometer (NanoDrop Technologies Inc., Wilmington, DE, USA). The RNA concentration of each sample was further examined using a Qubit® 2.0 Fluorometer (Life Technologies, CA, USA), and its quality was determined using an Agilent 2200 TapeStation (Agilent Technologies, Santa Clara, CA). RNA samples exhibiting integrity values of more than 7.0 was used for further RNA-seq library construction. The RNA sequencing library was conducted using the TruSeq® Stranded mRNA Sample Preparation Guideprovided by the manufacturer according to a previous study [25]. Briefly, total RNA was rRNA-depleted, fragmented, and used for the generation of cDNA via reverse transcription. The cDNA was then amplified by PCR, and the purified cDNA amplicons were subjected to sequencing at Génome Québec Center at McGill University (Montréal, Canada) using the HiSeq 2000 system (Illumina) to generate 100 bp paired-end reads.

### Transcriptome analysis

The high-quality sequencing reads were aligned to the reference genome (UMD 3.1/bosTau6, http://genome.ucsc.edu/) using Bowtie/TopHat2 according to a previous study [26]. Htseq counts were employed to filter the data to eliminate reads with more than two mismatches to the reference genome and reads showing multiple mapping hits [27]. Guided transcript assembly was conducted using the bovine reference genome assembly UMD3.1 (ftp://ftp.ncbi.nlm.nih.gov/genomes/Bos_taurus/website), in addition to the ENSEMBL reference annotation. Htseq counts were also used to count the reads that mapped to each of the bovine genes using the Tophat2 output [27]. The reads were normalized to counts per million (CPM) using the following formula: CPM = (number of reads mapped to a gene)/(total number of reads mapped to all annotated genes) × 10^6^. All the sequencing data (fastq files) generated in the present study are available in GEO database (http://www.ncbi.nlm.nih.gov/geo/) with accession number GSE78197.

### Identification of differentially expressed genes

The dietary effect on gene expression was investigated by characterizing dietary differentially expressed (DE) genes through pair-wise comparisons (AH vs. CS, AH vs. RS, and RS vs. CS) using the bioinformatics tool edge in R [28]. For each comparison, transcripts with CPM > 1 in at least 50% of the samples per treatment were subjected to DE analysis. Fold change (FC) was defined as the ratio of the arithmetic mean of the CPM within each comparison group, and differential expression was determined with log_2_FC > 1 or < -1 with the Benjamini-Hochberg method (FDR). Furthermore, the dietary associated DE genes were identified based on the mutually DE gene between every two dietary pair-wise comparisons. The mutually DE genes between AH vs. CS and AH vs. RS were identified as AH associated DE genes. Similarly, the mutually DE genes between CS vs AH and CS vs. RS were identified as CS associated DE genes, while the mutually DE genes between RS vs AH and RS vs. CS were identified as RS associated DE genes.

### Functional analysis

PANTHER (http://www.pantherdb.org/) was used to identify the functional classification of the biological processes that DE genes involved in. The enriched ontologies for DE genes were identified using web-based annotation tool DAVID v 6.8 (Database for Annotation, Visualization, and Integrated Discovery; https://david.ncifcrf.gov/) [29]. The DAVID-defined annotation selection was also employed to define Kyoto Encyclopedia of Genes and Genomes (KEGG) pathways and GO terms (GOTERM_XX) [30]. Clustering enrichment thresholds were set as required according to DAVID EASE scores. The classification stringency was set to medium, and the adjusted *P*-values obtained from Bonferroni correction for multiple testing [31] were used to define the significance of the identified functions.

### Validation of expression of selected DE genes using qRT-PCR

The total RNA of ruminal epithelium was reverse transcribed to cDNA using the PrimeScript 1st Strand cDNA Synthesis Kit (Code No. 6110A, Takara, Otsu, Japan). Quantitative real-time PCR (qRT-PCR) was performed with the 2 × SYBR® Premix Ex Taq (Tli RNaseH Plus) kit (Code No. RR420A, Takara, Otsu, Japan) and the Applied Biosystems 7500 (Foster City, CA). The PCR conditions were set as follows: 1 cycle at 95 °C for 10 min, 40 cycles of 95 °C for 15 s, and 60 °C for 34 s, followed by a melting curve program (60 to 95 °C). The primers (Table 1) targeting for *DSG1*, *PRSS53*, *BAG3*, *IGFBP3*, *CYR61*, *HSPB8*, *PI3*, *HLA-DQA1*, were designed using National Center for Biotechnology Information Nucleotide (http://www.ncbi.nlm.nih.gov/nuccore/), with *Bos taurus* set us species. The specificity of the primers was validated by primer-BLAST (https://www.ncbi.nlm.nih.gov/tools/primer-blast/). The *GAPDH* [32] and *β-actin* [33] were used as reference genes. The relative changes at the mRNA level for each individual gene was analyzed using the 2^-ΔCT^, ΔCT = CT_target mRNA_ – CT_house keeping mRNA_ (where CT = cycle threshold) method [34].

### Statistical analysis

The data on dietary physical factor, rumen fermentation characteristics, the WP, and relative mRNA expression using qRT-PCR were analyzed through pairwise comparisons using the PROC MIXED procedure in SAS (SAS 8.1) under a randomized complete block design, with diet as the fixed effect and the cows as the random effect. The results were reported as the least squares means. The statistical significance was declared at *P* ≤ 0.05, and the tendency was accepted at 0.05 < *P* ≤ 0.10.

The possible relationships between the expression of genes and the examined phenotypes (such as WP) were analyzed using one-way ANOVA and Pearson correlation using the whole dataset and the COR function in R software with diet as the fixed variable. Correlation analysis was firstly performed between all transcripts and VFA or WP, and then to determine how many of them overlapping with DE genes under each diet. These overlapped DE genes were then subjected a further correlation analysis under each diet. Significant positive or negative correlations were defined at *P* < 0.05.

## Results

### Nutritional and physical features of different forage based diets

The energy density and physically effective fiber differed among three diets. The peNDF_8.0_ was greater for RS compared with CS (*P* < 0.01) and AH (*P* < 0.01) (Table 2). The AH and CS had similar physical form, while AH was higher in energy density and CP content (*P* < 0.05). The CS and RS had similar energy density but different physical form with RS having a greater particle size. The AH had greater energy density but smaller particle size compared with RS. The AH diet had similar physical form but greater energy density in comparison with CS diet.

**Table 2:** Nutritional and Physical features [Particle size (PS) distribution (%) and physically effective NDF (peNDF)] of three experimental diets (*n* = 5)

	Diet^a^				*P*-value		
Item^b^	AH	CS	RS	SEM	AH vs. CS	AH vs. RS	CS vs. RS
Nutritional factor							
CP	16.7	16.2	16.0	0.25	0.02	0.01	0.47
Net energy for lactation_,_ Mcal/kg	1.57	1.45	1.43				
Physical factor							
PS, >19.0mm	32.2	16.9	40.4	1.84	<0.01	0.03	<0.01
PS, 8.0 to 19.0 mm	16.0	30.6	10.4	1.89	<0.01	0.07	<0.01
PS, 1.18 to 8.0 mm	35.8	36.1	33.0	2.08	0.94	0.46	0.18
PS, <1.18 mm	16.1	16.4	16.2	1.34	0.81	0.96	0.91
peNDF_19.0_	10.0	6.1	14.9	0.67	<0.01	<0.01	<0.01
peNDF_8.0_	23.4	25.0	33.7	1.71	0.34	<0.01	<0.01

^a^AH = Total mixed ration (TMR) containing alfalfa hay as the main forage; CS = TMR containing corn stover as the main forage; RS = TMR containing rice straw as the main forage

^b^CP = crude protein; NE_L_ = Calculated values based on Chinese recommondations; NDF = neutral detergent fiber; peNDF_8.0_ measured as the NDF content of diet multiplied by proportion of particles > 8.0 mm; peNDF_19.0_ measured as the NDF content of diet multiplied by proportion of particles > 19.0 mm; Particle size distribution determined by the Penn State Particle Separator sieving technique

### Rumen fermentation characteristics

Total VFA concentration was greater in the AH-fed cows compared with the cows consumed CS (*P* = 0.04) or RS (Table 3, *P* = 0.02), respectively. No difference was observed between the cows fed RS and CS (*P* = 0.63). The acetate concentration was greater in AH-fed cows than in cows fed CS (*P* = 0.03) or in RS (*P* = 0.02, Table 3). The propionate concentration tended to be higher in cows fed AH compared with those fed RS (*P* = 0.07). The ratio of acetate to propionate tended to be higher in cows fed RS compared with those fed CS (*P* = 0.07), while there was no difference between the AH and RS animals. The butyrate concentration was lower in RS-fed cows than in CS- or AH-fed cows (*P* = 0.02), whereas the molar proportion of butyrate was greater in the CS-fed cows than in AH-fed cows (*P* < 0.01) or RS (*P* = 0.02).

**Table 3:** Rumen fermentation characteristics in dairy cows fed with three experimental forage-based diets (*n* = 6)

	Dietary treatments^a^				*P*-value		
Item	AH	CS	RS	SEM	AH vs. CS	AH vs. RS	CS vs. RS
pH	6.39	6.41	6.39	0.050	0.89	0.98	0.85
Ammonia-N, mg/dL	14.5	15.8	20.3	1.07	0.37	0.01	0.04
Total VFA, m*M*	93.5	81.0	83.1	2.83	0.04	0.02	0.63
Acetate, m*M*	66.2	57.1	59.8	1.99	0.03	0.02	0.20
Propionate, m*M*	19.4	17.2	17.1	0.75	0.14	0.07	0.95
Butyrate, m*M*	7.90	8.64	6.18	0.483	0.13	0.09	0.02
Molar proportion, %							
Acetate	71.1	70.3	72.2	0.89	0.03	0.40	0.02
Propionate	20.7	21.4	20.5	0.55	0.51	0.80	0.36
Butyrate	8.29	10.9	7.34	0.556	<0.01	0.27	0.02
Acetate: Propionate	3.47	3.24	3.60	0.108	0.23	0.45	0.07

^a^AH = Total mixed ration (TMR) containing alfalfa hay as the main forage; CS = TMR containing corn stover as the main forage; RS = TMR containing rice straw as the main forage

### Morphological parameters of ruminal papillae

**Fig. 2:**
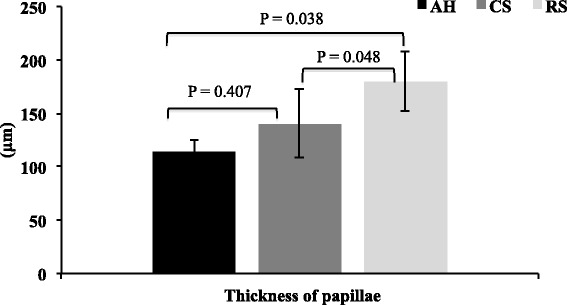
The evaluation of width of rumen papillae in dairy cows fed three different diets (*n* = 6). AH = Total mixed ration (TMR) containing alfalfa hay as the main forage; CS = TMR containing corn stover as the main forage; RS = TMR containing rice straw as the main forage

The cows fed RS had a thicker ruminal papilla layer (Fig. 2) than the cows fed AH (*P* = 0.038) or CS (*P* = 0.048). Whereas WP was not changed between the cows fed AH or CS diet (*P* > 0.05).

### Transcriptome profiling of rumen epithelial tissues

In total, 721.2 M reads were generated from 18 samples, ranging from 35.2 to 53.6 M per sample. Among them, 593.8 M reads were mapped to the bovine genome, ranging from 28.2 to 47.9 M per sample. Totally, the expression of 12,943, 12,935, and 13,020 genes was detected in the rumen epithelia and 102, 83, and 131 of them expressed uniquely when cows fed AH, CS and RS diets, respectively (Additional file 1: Figure S1-A). The predominant 2490 genes covered more than 80% abundance of total mapped reads (Additional file 1: Figure S1-B), with predominant functions in translation, ribosomal protein, and structural molecule activity.

### Dietary effects on gene expression in rumen papillae

A total of 31, 40, and 28 DE genes were identified from the comparison of AH vs. CS, AH vs. RS, and RS vs. CS, respectively (FDR < 0.05, Additional file 2: Table S1). Among DE genes identified in AH vs. CS, 13 of them were up-regulated (log_2_FC ranged from 1.01 to 2.99), while 18 genes were down-regulated (log_2_FC ranged from -3.25 to -1.18). Similarly, 22 and 24 DE genes from the comparison of AH vs. RS (log_2_FC ranged from 1.01 to 2.45). and RS vs. CS (log_2_FC ranged from 1.02 to 2.82), respectively, were up-regulated, while 18 (log_2_FC ranged from -3.12 to -1.01) and four (log_2_FC ranged from -1.87 to -1.02) of them were down-regulated respectively.

### Function and pathway analysis of differentially expressed genes under different diets

**Fig. 3:**
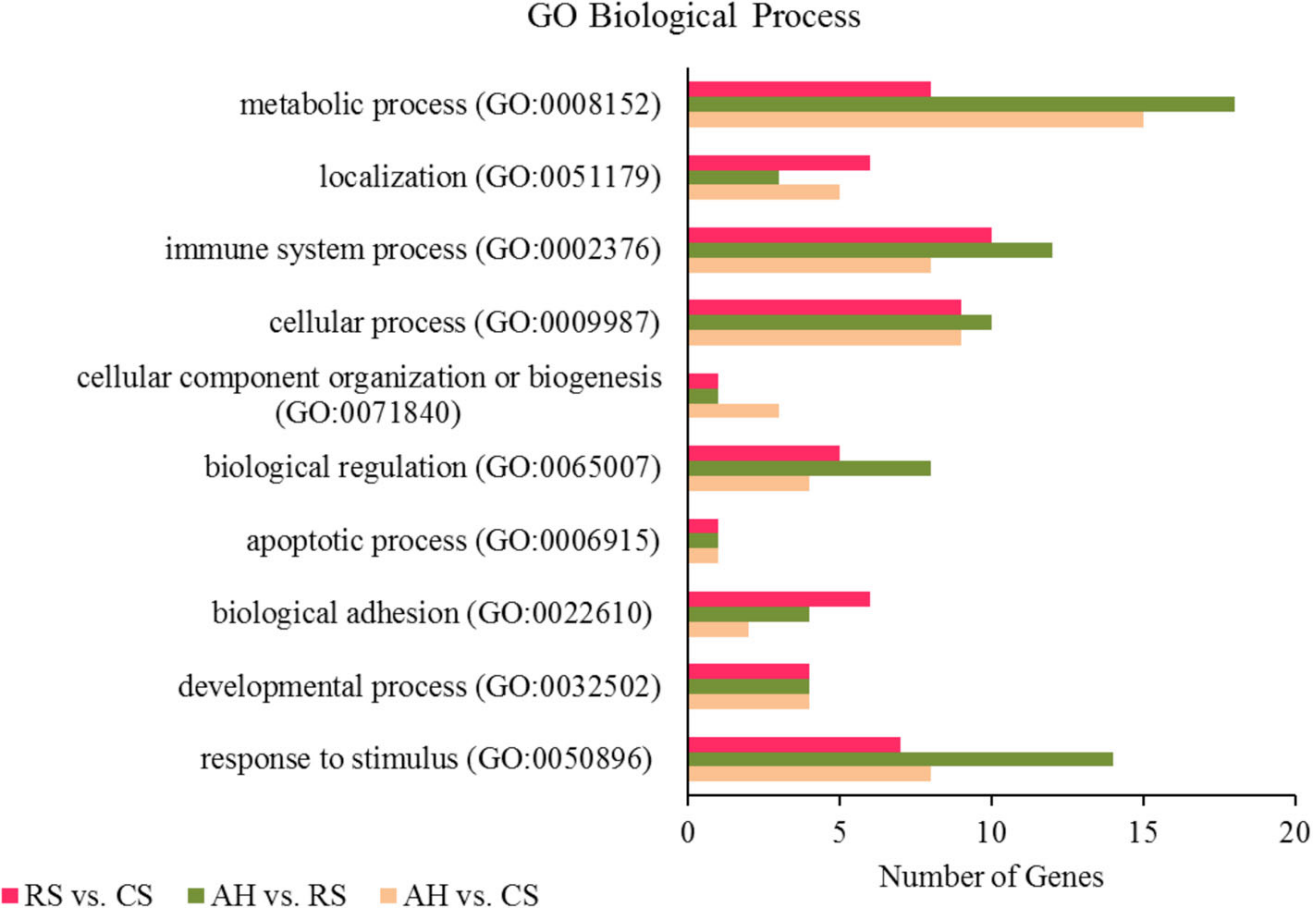
Functional classification involved in biological process targeted by dietary DE genes in the rumen of dairy cows fed three different diets. AH = Total mixed ration (TMR) containing alfalfa hay as the main forage; CS = TMR containing corn stover as the main forage; RS = TMR containing rice straw as the main forage

The functional analysis using DAVID revealed that DE genes under three dietary comparisons were involved in different functions (Table 4). The identified DE genes including *DSG1*, *EFEMP1*, *S100A9*, *LPO*, *PADI1*, *S100A8*, *AIF1*, *RCN1*, and *TGM3*, from AH vs. CS were involved in calcium ion binding (*P* = 2.0E-4), while identified DE genes including *C3*, *C7*, and *CFH* from RS vs. CS were involved in complement activation (*P* = 4.8E-3). The DE genes including *S100A9*, *APOLD1*, *C2*, *CFB*, *CRISP3*, *LAP*, *PI3*, *SLURP1*, and *TAP* between AH and RS, were predicted to impact extracellular region (*P* = 5.9E-4). The functional classification determined using PANTHER showed that the DE genes were mainly involved in of the functions of stimulus response, developmental processes, biological adhesion, apoptotic processes, biological regulation, cellular component organization or biogenesis, cellular processes, immune system processes, localization, and metabolic processes, with different numbers of DE genes for the three pair-wise comparisons (Fig. 3).

**Table 4:** Enriched functions of dietary differentially expressed genes based on GO terms analyzed using DAVID

Functional term	Count	%	*P*-value	*P*-value (adjusted)	Fold Enrichment	Involved genes
*AH vs. CS*						
calcium ion binding	9	29.0	5.3E-6	2.0E-4	8.2	*DSG1, EFEMP1, S100A9, LPO, PADI1, S100A8, AIF1, RCN1, TGM3*
*AH vs. RS*						
extracellular region	9	22.5	1.3E-5	5.9E-4	7.7	*S100A9, APOLD1, C2, CFB, CRISP3, LAP, PI3, SLURP1, TAP*
*RS vs. CS*						
complement activation, alternative pathway	3	10.7	5.1E-5	4.8E-3	256.9	*C3, C7, CFH*

^1^AH = Total mixed ration (TMR) containing alfalfa hay as the main forage; CS = TMR containing corn stover as the main forage; RS = TMR containing rice straw as the main forage

The KEGG pathways of DE genes were analyzed among the three pair-wise comparisons. Only one KEGG pathway, complement and coagulation cascades, was enriched (*P* < 0.01; Additional file 3: Figure S2) in the comparison between RS and CS.

### Diet-associated DE genes

**Fig. 4:**
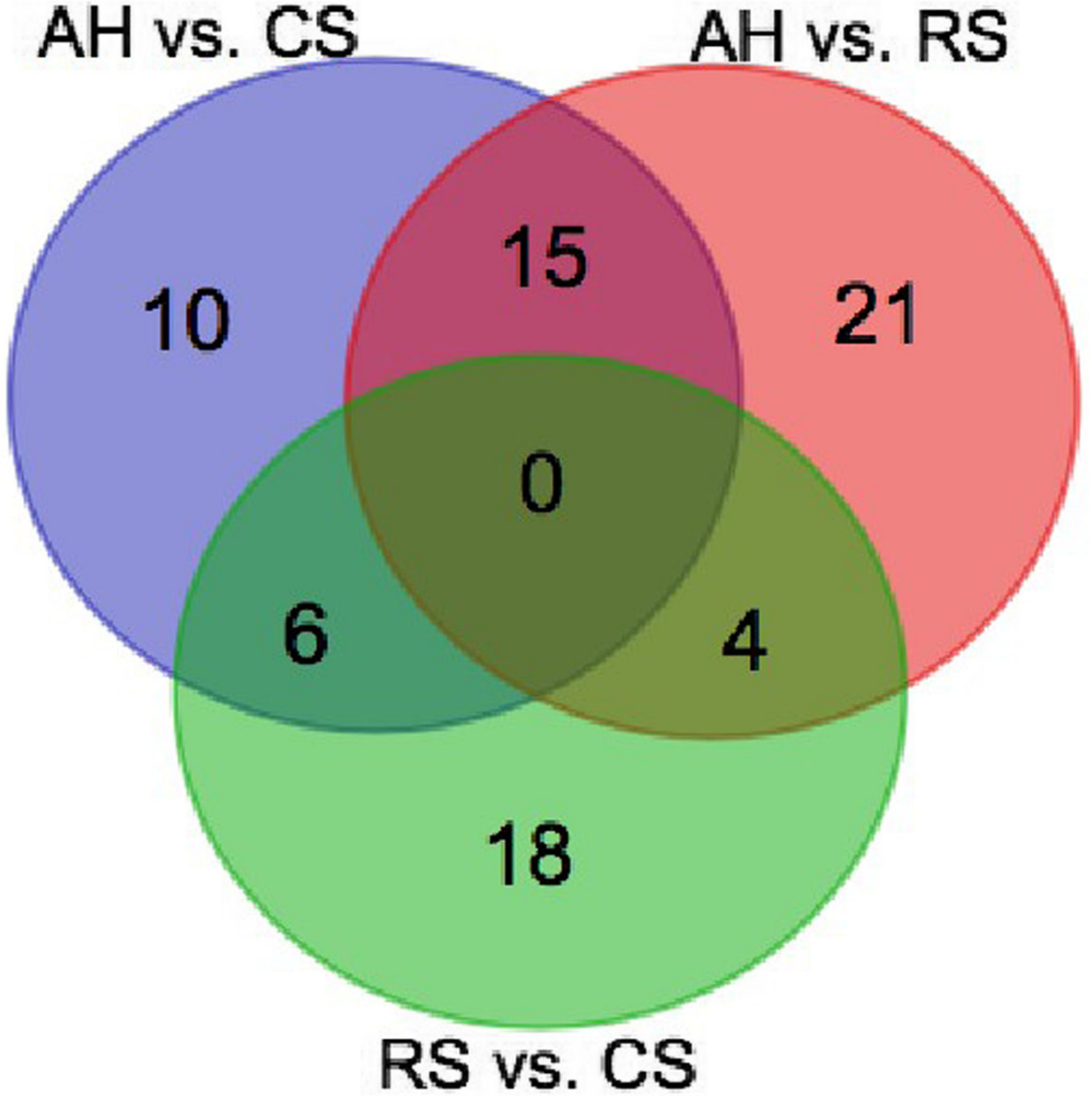
The dietary associated differentially expressed (DE) genes. AH associated DE genes, the mutually DE genes between AH vs. CS and AH vs. RS; CS associated DE genes, the mutually DE genes between CS vs AH and CS vs. RS; RS associated DE genes, the mutually DE genes between RS vs AH and RS vs. CS. AH = Total mixed ration (TMR) containing alfalfa hay as the main forage; CS = TMR containing corn stover as the main forage; RS = TMR containing rice straw as the main forage

According to Venn diagrams analysis, the dietary associated DE genes were further identified (Fig. 4). A total of 15, 6, and 4 genes were identified as AH associated DE genes, CS associated DE genes, and RS associated DE genes, respectively. Among AH associated DE genes, the expression of *PI3*, *AIF1*, *HSPB8*, *BAG3*, and *PRSS53* were down-regulated, while the expression of *DSG1* and *ARG1* were up-regulated. Similarly, for CS associated DE genes, the expression of *MT-ATP8* and *EFEMP1* were down-regulated, while the expression of RS associated DE genes *HLA-DQA1* and *HLA-DQB1* were up-regulated.

### Association between ruminal fermentation characteristics and dietary DE genes

The relationships between ruminal fermentation characteristics and DE genes were further explored using correlation analysis. In total, the expression of 12 genes were significantly correlated with at least one of the fermentation parameters (VFAs) regardless of diet, with three of them (*C3, C7,* and *IFI47*) positively correlated with butyrate concentration (*P* < 0.05) and nine of them (*C2*, *Gm8618*, *KRT36*, *LPO*, *PI3*, *PRSS53*, *S100A9*, *SAA1*, and *TGM3*) negatively correlated with at least one of the fermentation parameters (*P* < 0.05) (Table 5). Among them, *C7* and *SAA1* were DE and their expressions were correlated with the concentration of total VFA and acetate, propionate respectively, under AH. Similarly, *LPO* and *PI3* were DE and correlated with total VFA, acetate, and propionate, and DE *KRT36* was correlated with the four characteristics under CS. Under RS, the expression of *C2* (DE) was correlated with the concentration of total VFA and acetate (Table 5).

**Table 5:** Correlations between the concentration of volatile fatty acids and expression of detected genes (*P* < 0.05, *n* = 18 vs. *n* = 6) in dairy cows fed with three experimental forage-based diets

Genes	Total VFA	Acetate	Propionate	Butyrate
All^a^				
*C2*	−0.51	−0.61		
*C3*				0.54
*C7*				0.47
*Gm8618*		−0.49		
*IFI47*				0.54
*KRT36*	−0.52		−0.48	−0.60
*LPO*	−0.52			−0.67
*PI3*	−0.56	−0.55		
*PRSS53*	−0.63	−0.62	−0.48	
*S100A9*	−0.61	−0.64	−0.48	
*SAA1*	−0.52	−0.54	−0.52	
*TGM3*	−0.52	−0.53		
AH^b^				
*C7*	0.88	0.88		
*SAA1*			−0.91	
CS^c^				
*LPO*	−0.95	−0.92	−0.88	
*PI3*	−0.91	−0.88	−0.86	
*KRT36*	−0.93	−0.84	−0.94	−0.88
RS^d^				
*C2*	−0.86	−0.86		

^a^All, based on analysis with all 18 animals

^b^AH = Total mixed ration (TMR) containing alfalfa hay as the main forage

^c^CS = TMR containing corn stover as the main forage

^d^RS = TMR containing rice straw as the main forage

### Association between rumen epithelial morphology phenotype and dietary associated DE genes

We further investigated the correlations between morphological traits (WP) and gene expression (Table 6). In total, the expressions of two genes were significantly correlated with WP regardless of diet: *HLA-DQA1* (*R* = 0.52, *P* < 0.05) positively correlated with WP, and *UGT2B17* (*R* = -0.69; *P* < 0.01) negatively correlated with WP. Among them, the DE gene *HLA-DQA1* was also correlated with WP under RS diet (*R* = 0.94; *P* < 0.01), and *UGT2B17* was also correlated with WP under CS (*R* = -0.94; *P* < 0.01) and RS diet (*R* = -0.96; *P* < 0.01).

**Table 6:** Correlation between the rumen morphology and the expression of differential expressed genes (*P* < 0.05, *n* = 18 vs. *n* = 6) in dairy cows fed with three experimental forage-based diets

Item	R	Item	R
All^a^		CS^b^	
*HLA-DQA1*	0.52	*UGT2B17*	−0.92
*UGT2B17*	−0.69	RS^c^	
		*HLA-DQA1*	0.94
		*UGT2B17*	−0.96

^a^All, based on analysis with all 18 animals

^b^CS = Total mixed ration (TMR) containing corn stover as the main forage

^c^RS = TMR containing rice straw as the main forage

### Quantitative real-time PCR validation of expression of ruminal morphology associated genes

**Fig. 5:**
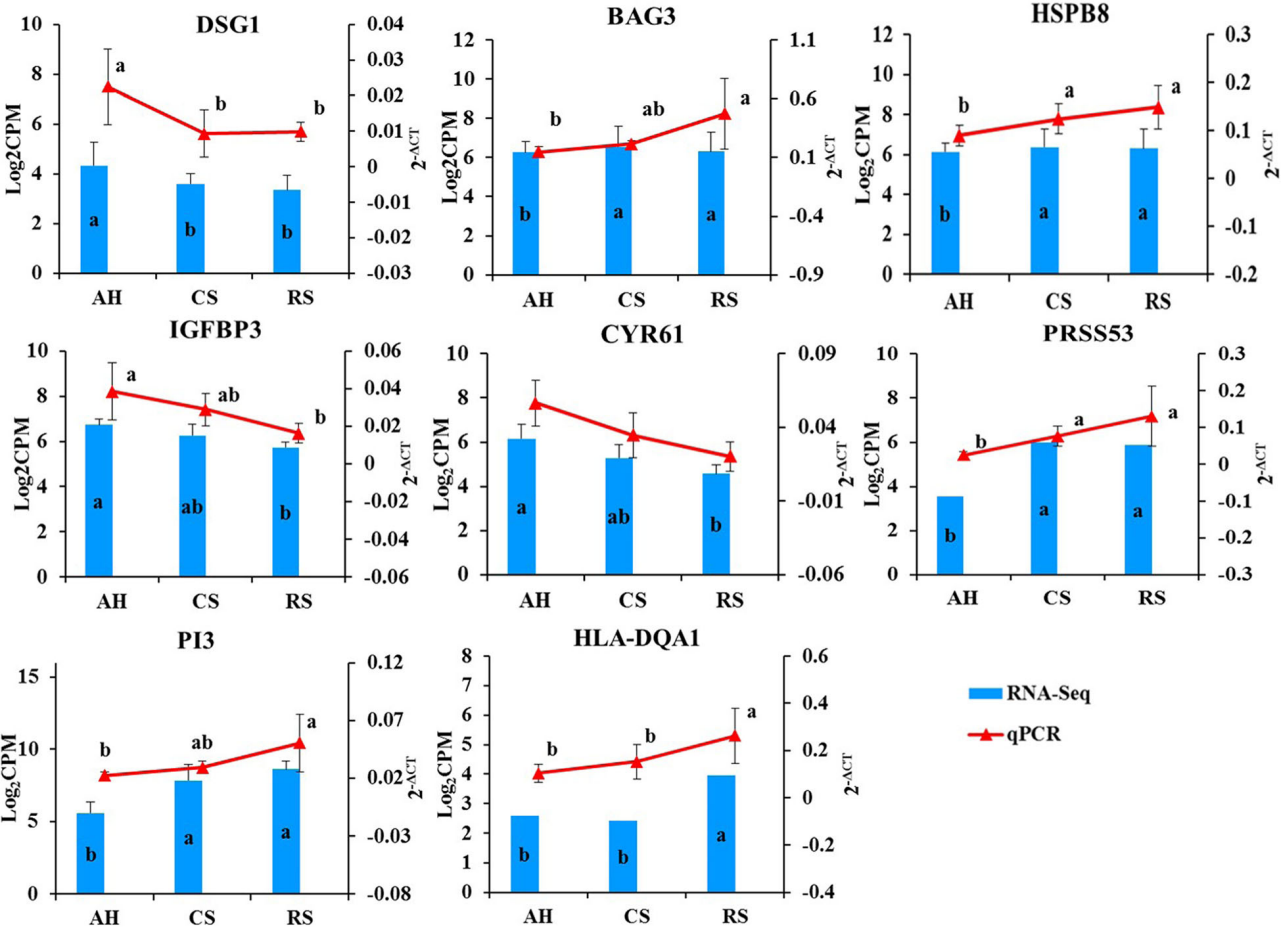
Expression of *DSG1*, *PRSS53*, *BAG3*, *IGFBP3*, *CYR61*, *HSPB8*, *PI3*, and *HLA-DQA1* detected by quantitative real-time PCR validation and RNA-seq. AH = Total mixed ration (TMR) containing alfalfa hay as the main forage; CS = TMR containing corn stover as the main forage; RS = TMR containing rice straw as the main forage. Note: a-b means within same index column with different superscripts differ (*P* < 0.05; among the three pair-wise comparisons)

The DE genes including *DSG1*, *PRSS53*, *BAG3*, *IGFBP3*, *CYR61*, *HSPB8*, *PI3*, and *HLA-DQA1* were selected for qPCR validation (Fig. 5). The qPCR analysis confirmed that the gene expression of *HLA-DQA1, HSPB8*, *PRSS53*, *BAG3*, and *PI3* was higher (*P* < 0.05) in the rumen epithelial tissue of cows fed with RS, while the expressions of *DSG1*, *IGFBP3*, and *CYR61* (tended, *P* = 0.052) were lower in the RS compared with AH. The expressions of *PRSS53* (*P* < 0.05), *HSPB8* (*P* < 0.05), and *BAG3* (tended; *P* = 0.053) and *PI3* (tended; *P* = 0.066) were greater in cows fed CS, compared with those consumed AH, while the expression of *DSG1* was lower in CS compared with that in AH fed cows (*P* < 0.05). Additionally, the expression of *HLA-DQA1* was greater in RS fed cows compared with CS-fed cows (*P* < 0.05).

## Discussion

RNA-seq is an effective, efficient, and widely used method for conducting analyses in transcriptional status and functional gene discovery [7, 14, 16]. The transcripts detected from rumen epithelial tissue in this study with dairy cows were relatively lower compared with the previous study detected from rumen epithelial tissue of beef cattle [35] (12,868 vs. 14,709 transcripts). However, the majority of the transcripts was identified in both studies, with 12,516 common transcripts. In this study, feeding lactating dairy cows with three diets with different forage sources, which varied in feed particle sizes and energy densities, led to changes in rumen papillary morphology and VFAs concentrations in the rumen. By linking dietary composition, rumen fermentation traits, rumen epithelial morphology, and rumen epithelial transcriptomes together, the effects of dietary physical and nutritional factors on rumen epithelial morphology and the underlying mechanisms were identified.

### Nutritional factors

In the current study, we found that the function of AH associated DE gene *DSG1* (up-regulated in cows fed AH, compared with those fed CS and RS) was enriched under the ion-binding function as well as was associated with tight junctions. This gene encodes a member of the desmoglein protein subfamily which has been reported to be involved in epithelial cell proliferation in bovine epidermis and tongue papillae [36]. It has been speculated that ion binding in the rumen may be attributed to the Na^+^-K^-^-ATPase transport system for nutrient absorption [37]. Na^+^-K^-^ pumps are predominately localized to the innermost living cell layers of the stratum spinosum and the stratum germinativum [38]. Increased VFAs absorption by greater Na^+^-K^-^-ATPase activity could prevent possible disturbance of epithelial functions (transport and barrier functions) [39]. The higher amount of VFAs and the greater expression of *DSG1* in cows fed AH diet indicates likely more nutrient transport through RE when animals fed AH diet.

In the meantime, the absorption of the nutrients through the RE consists of active transport and passive transport pathways [40]. The transmural movement of VFAs is a concentration-dependent passive diffusion process, which contributes to the absorption of VFA in addition to the carrier-mediated VFAs transport [41]. The rate of uptake of VFAs through passive pathway can be as high as up to 75% in the RE [42], and the WP of RE could be directly associated with the passive nutrient uptake. Therefore, the increased WP in RS fed animals indicate the potentially lower absorption of VFA which may lead to lower productivity and feed efficiency [18] when compared to AH and CS-fed animals. Moreover, the function of *ARG1* (higher expression in AH compared with CS or RS) has been reported to be involved in catalyzing the hydrolysis of arginine to ornithine and urea in bovine liver [43]. It has been reported that N conversion efficiency in CS or RS-fed cows were lower than that in AH-fed cows [18]. The observed expression differences in genes involved in AA metabolism in RE may further provide the explanation of RE function in N metabolism under different forage-based diets.

In this study, DE gene *C7* (lower expression in CS compared with AH or RS) was enriched in the function of complement activation, alternative pathway, playing an integral role in the assembly of complement complex [44], and *C2*, an activator of the classical complement pathway [44], was enriched in the function of extracellular region. Activation of the complement system forms a major part of the innate immune system, resulting in the formation of complement complex [44]. Complement C2/C7 was involved in the complement component that is a key system for immune surveillance and homeostasis [45]. Serum amyloid A protein (SAA) is an inflammatory factor and plasma SAA is considered as the important acute phase proteins in cattle [46]. Furthermore, the expression of *C2*, *C7*, and *SAA1* was correlated with the concentration of butyrate (positively), total VFA and acetate (negatively), and total VFA, acetate, and propionate (negatively), respectively, regardless of dietary effects. To date, the expression of these genes was not reported from RE, and the findings of its relationship with butyrate and other VFAs under AH or RS may provide the evidence on its function in the rumen health.

In addition, the expression of *LPO* and *KRT36* was negatively correlated with butyrate and total VFA concentration regardless of dietary effects or in CS. Lactoperoxidase (LPO) is an important component of defense mechanism in the body with the antimicrobial and physical properties [47]. Keratins (KRT), the epithelial-specific intermediate filaments, changes in keratin gene expression implied the effect on terminal keratin synthesis, which might affect the epithelium morphology [48]. The previous study found that butyrate infusion could down-regulate the expression of *LPO* and *KRT36* in RE [7]. Moreover, the expression of *PI3* was lower in cows fed AH compared with CS and RS and enriched in the function of extracellular region. The *PI3* gene, which is also known as the elastase-specific inhibitor, is a low-molecular-weight proteinase inhibitor that is synthesized and secreted at the site of neutrophil infiltration [49]. The covalent cross-linking of the *PI3* protein to ECM proteins [50], confers the antimicrobial activity on *PI3* and promotes its involvement in the innate immune system, and protects epithelial surfaces from being infected [51]. Therefore, the down-regulation of *PI3* together with increased VFAs in AH may suggest the protective function against for adverse or irritant factors’ absorption but promoting VFAs’ assimilate from RE, which then improving the animal performance for AH-fed animals. Therefore, the identified DE genes and their relationships with VFAs in response to different forage diets reveal that the observed changes in RE morphology could result from dietary induced VFA changes, especially butyrate, and their effect on gene expression. Future studies are needed to understand the roles of VFAs on the expression of *C2*, *C7*, *SAA1, LPO, KRT36, PI3* and rumen epithelium barrier, nutrient absorption, as well as the pathway of complement and coagulation cascades in RE using *in vitro* and *in vivo* models.

In ruminants, the stimulation of papilla growth by VFAs or concentrates stimulating rumen epithelial functions has also been well described [9], for example, butyrate is a specific inhibitor of ruminal apoptosis as well as a stimulator of papillae proliferation [11]. Additionally, the cellular or tissue proliferation/apoptotic processes in RE could directly affect rumen papillae morphology. In this study, we reported the expression of *BAG3* was down-regulated in cows fed AH and was positively correlated with ruminal WP (Table 6). The *BAG3* has been reported to play an important role in cell proliferation, apoptosis, migration, and invasion [52], suggesting that the lower expression of this gene could be associated with the lower proliferation/apoptotic processes in RE which could subsequently lead to thinner epithelial layer in cows fed AH. In addition, it has been reported that *BAG3* and *HSPB8* (Heat shock protein family B (small) member 8) were chaperone complex targeting misfolded proteins to macroautophagy [53], which is a process that can be activated in response to the nutrient deficiency in eukaryotic cells [54]. HSPB8 is known to recognize the misfolded proteins whereas BAG3 might recruit and activate the macroautophagy machinery [53]. Thus, the expression differences in *HSPB8* and *BAG3* in cows fed CS and RS suggest that the cells of RE might suffer macroautophagy due to the lower energy supply and nutrient availability (VFAs) from these two cereal straw diets.

From the above results, it is suggested that energy density may regulate the immunologic barrier and nutritional transport in ruminal epithelium by influencing ion binding, complement activation, and cell growth, partly induced by altered ruminal VFAs concentration.

### Physical factors

Recent studies have proposed that in addition to nutrients (VFAs or concentrates), fiber particle characteristics (size) and the effective fiber content may also play a key role in rumen muscularization and volume development [55]. In the current study, the observed significant greater rumen papillae width when animals fed with RS, which contains higher peNDF comparing to in CS and AH, further highlights the importance of dietary physical factor in affecting the ruminal morphology.

Particle size can regulate rumen development by altering blood supply to the RE [4]. When cows ingested large quantities of solid rice straw, the RE was exposed to indigestible solid fiber, and the rumen cyclically contracted to mix the solid fiber [56]. Accordingly, the cows fed a diet with a larger particle size (RS diet) may secrete more saliva, since the salivary secretion could be enhanced by an increased intake of physically effective fiber [57]. It has been proposed that rumen development regulation can be mediated through decorin (DCN) and the EGF receptors and that a direct interaction exists between these two receptor to regulate cell growth during remodeling [58]. The EGF, a bioactive peptide [59], stimulates epithelial proliferation and differentiation when supplied to the luminal side of the rumen via the saliva [60]. Intrinsic EGF-like molecules in the digestive tract are known to maintain mucosal integrity by controlling epithelial cell turnover and accelerating the healing of mucosal injuries [61]. Indeed, the expression of gene encoding multiple EGF like domains 8 (*MEGF8*) was positively correlated with the rumen papillae width (*P* = 0.01; *R* = 0.58) although it was not differentially expressed among the diets. Gene expression is not always correlated with the protein expression, therefore, future study is needed to measure the protein level of DCN and MEGF8, in order to provide more information on how these two genes are involved in rumen epithelial cell proliferation. In addition, the expression of *DCN* (decorin) was significantly higher in RS compared to CS-fed animals, which might be attributed to the increase in salivary secretion due to stimulation by a larger dietary particle size [62]. Future studies are needed to measure the salivary secretion difference in AH, RS, and CS-fed cows to verify their role in affecting WP.

In the current study, the expression of *IGFBP3* was lower in cows fed RS compared with those fed AH. The *IGFBP3* has been well characterized to have functions opposing IGF-1 events [63]. IGF-1 is reported to be involved in rumen epithelial growth, as it induces RE cell proliferation *in vitro* [7]. The cellular events associated with IGF-1 are modulated by the function of IGFBP [64], which may either inhibit or stimulate cellular proliferation or differentiation due to the effects of IGFs [65]. In addition, the down-regulated expression of the *IGFBP3* genes in RE of cows was mainly due to the accelerated cellular migration and postmitotic aging induced by subacute ruminal acidosis, which can influence ruminal epithelial growth [66]. The changes in mRNA abundance of *IGFBP3* in RS cows might be attributed to reduced organization and intercellular adhesion between the cells of the stratum granulosum. This suggests that the physical factor affects the structural integrity of the RE, through influencing the expression of several related genes such as *IGFBP3*. In the meantime, the expression of *CYR61* and *NOV* were also down-regulated in RS fed cows compared to that in AH fed cows. Their encoded proteins are members of the *CNN* family, which share similarities with *IGFBP. CYR61* is a secreted, extracellular matrix (ECM)-associated heparin-binding protein, mediating cell adhesion, stimulates cell migration, and potentiates growth factor-induced DNA synthesis in fibroblasts and endothelial cells [67]. Therefore, the down-regulation of *IGFBP3* and *CYR61*, as well as up-regulation of *PI3* might enhance cellular growth in the RE under the RS diet, which could be one of the causes of the increase in epithelial width.

The expression of *HLA-DQA1* was higher in cows fed RS, compared to CS, and was significantly positively correlated with the ruminal WP. It has been reported that the down-regulation of this gene can reduce antigen-specific human T cell proliferation [68], which might indicate the potential function of *HLA-DQA1* in cell proliferation and explain the observed relationship with rumen epithelial width under RS diet. The expression of *UGT2B17* was down-regulated in cows fed RS diet compared with that fed CS diet. Down-regulation of *UGT2B17* by growth factors such as fibroblast growth factor family may increase the proliferation of androgen-dependent tumors [69]. Therefore, its significant negative correlation with the WP might indicate its potential function in the proliferation of rumen epithelium. Although both *HLA-DQA1* and *UGT2B17* showed potential relationship with WP under different diets, the functions of these genes in the rumen epithelia have not been reported. Future studies are needed to verify their changes in expression and their function in cell proliferation in RE, and how they could be related to rumen WP in other studies or potentially *in vitro* cell models. Regardless, this is the first study to report the physical factor derived expression change of these genes in the rumen epithelia and their relationship with rumen epithelial morphology. Future studies are needed to verify their function and the action of mode in impacting the ruminal epithelial morphology.

## Conclusion

**Fig. 6:**
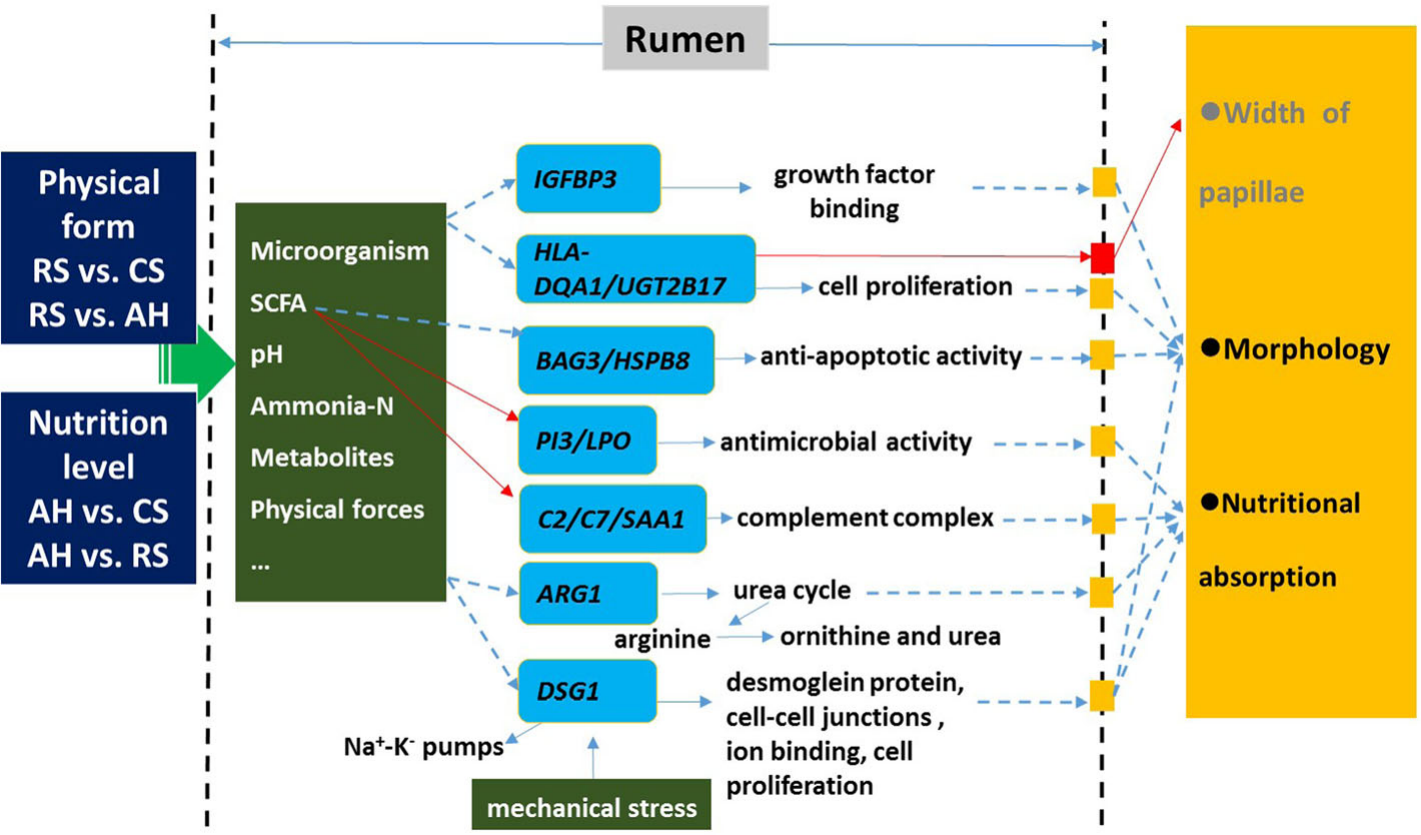
A proposed relationship between transcriptional mechanism and phenotype of the morphology of RE affected by nutritional and physical factors in the cows fed three different diets. The arrows with solid red line indicate the significant correlation between the two defined items from this study (*P* < 0.05). Arrows with solid blue line indicate the determined or reported relationship between the two defined items. While arrows with dotted blue line indicate the potential correlation between the two items identified from this study. AH = Total mixed ration (TMR) containing alfalfa hay as the main forage; CS = TMR containing corn stover as the main forage; RS = TMR containing rice straw as the main forage. The different colors or shapes across the genes represent the differentially expressed genes among the comparisons between 2 dietary treatments. *BAG3* = BCL2-associated athanogene 3; *C2* = complement C2; *C7* = complement C7; *DSG1* = desmoglein 1; *HLA-DQA1* = major histocompatibility complex, class II, DQ alpha 1; *HSPB8* = heat shock protein family B (small) member 8; *IGFBP3* = insulin-like growth factor binding protein 3; *LPO* = lactoperoxidase; *PI3* = peptidase inhibitor 3, skin-derived; *SAA1* = serum amyloid A1; *UGT2B17* = UDP glucuronosyltransferase family 2 member B17. Color version available in the online PDF

In summary, our obtained DE genes by transcriptome analysis suggest that diet can affect the expression of genes involved in ion binding, cellular growth, and cell proliferation, which may lead to the alteration of RE morphology. A possible mechanism that affects the rumen epithelium function and morphology through physical and nutritional factor under different forages based diets was proposed (Fig. 6). The interaction of dietary nutritional and physical factors could affect the rumen microbiota and microbial fermentation that could subsequently alter the expression of the DE genes including *IGFBP3*, *C2*, *C7*, *SAA1*, *BAG3, HSPB8, PI3*, *LPO*, *ARG1*, *DSG1*, *HLA-DQA1,* and *UGT2B17*, which have the function associated with the tissue morphology or nutritional absorption. The DE genes *HLA-DQA1* and *UGT2B17* identified in this study may be also involved in cell proliferation in rumen epithelium, with further validation research needed. Among the identified DE genes, *HLA-DQA1*, *IGFBP3*, *DSG1*, and *BAG3* may be potential gene markers for the RE morphology and the indicators for nutrient metabolism in the rumen, *C7* and *PI3* may be potential gene markers for the nutrient transport such as VFAs absorption through RE. Overall, our results suggest that the dietary physical form is important for the morphology of RE in lactating dairy cows in addition to the dietary nutritional level, indicating that the forage particle size should be moderate when prepared for total mixed ration diet; and that shorter particle size of low-quality forage may be beneficial for VFAs synthesis as well as absorption (especially through passive transport). Future studies are needed to identify how the morphological changes in the RE-induced by nutritional and physical factors as well as the interaction between them impact nutrient absorption by the rumen papillae, thereby increasing our understanding of how feeding strategies influence the function of the rumen. Also, measuring the protein level of the identified DE genes from this study is needed to verify the dietary effects on the relationship between the RE morphology and altered gene expression. Regardless, our results provide novel insights into the potential transcriptional mechanisms involved in regulating rumen epithelial width and may offer guidance for future experimentation related to balancing rumen epithelial characteristics and dietary regimens.

## Additional files

Additional file 1:Figure S1. The identified genes using RNA-seq in rumen under AH, CS and RS diets (A) and the predominant 2490 genes (B; covered more than 80% abundance of total mapped reads). (PDF 41 kb)

Additional file 2: Table S1. Differentially expressed genes identified from the comparison of AH vs. CS, AH vs. RS, and RS vs. CS, respectively. (DOC 160 kb)

Additional file 3: Figure S2. The enriched pathway through the DE genes in the comparison between CS and RS. CS = Total mixed ration (TMR) containing corn stover as the main forage; RS = TMR containing rice straw as the main forage. (PDF 357 kb)

## Abbreviations

AH: Alfalfa hay based diet
*ARG1*: Arginase 1
*BAG3*: Athanogene 3
*C2*: Complement C2
*C7*: Complement C7
CPM: Counts per million
CS: Corn stover based diet
*CYR61*: Cysteine-rich, angiogenic inducer, 61
*DCN*: Decorin
DE: Differentially expressed
*DSG1*: Desmoglein 1
ECM: Extracellular matrix
*EFEMP1*: EGF-containing fibulin-like extracellular matrix protein 1
EGF: Epidermal growth factor family
FDR: Benjamini-Hochberg method
*HLA-DQA1*: Major histocompatibility complex, class II, DQ alpha 1
*HLA-DQB1*: Major histocompatibility complex, class II, DQ beta 1
*HSPB8*: Heat shock 22kDa protein 8
*IGFBP3*: Insulin-like growth factor binding protein 3
KEGG: Kyoto Encyclopedia of Genes and Genomes
*LPO*: Lactoperoxidase
*MT-ATP8*: Skin-derived; mitochondrially encoded ATP synthase 8
NDF: Natural detergent fiber
*NOV*: Nephroblastoma overexpressed
peNDF: Physically effective NDF
*PI3*: Peptidase inhibitor 3
RE: Rumen epithelium
RNA-seq: RNA sequencing
RS: Rice straw based diet
*SAA1*: Serum amyloid A1
TGF-α: Transforming growth factor-α
TGF-β1: Transforming growth factor-β1
TMR: Total mixed ration
*UGT2B17*: UDP glucuronosyltransferase family 2 member B17
VFA: Volatile fatty acid
WP: Width of rumen papillae
