# Supplementary figures and images for: Effects of dietary physical or nutritional factors on morphology of rumen papillae and transcriptome changes in lactating dairy cows based on three different forage-based diets
Source: BMC Genomics. 2017 May 6;18:353. doi: 10.1186/s12864-017-3726-2 (PMC5420399; doi:10.1186/s12864-017-3726-2)

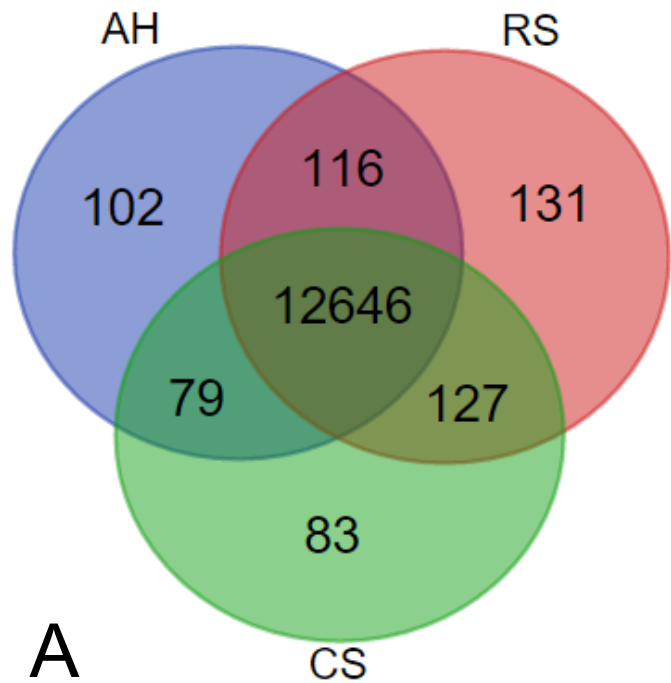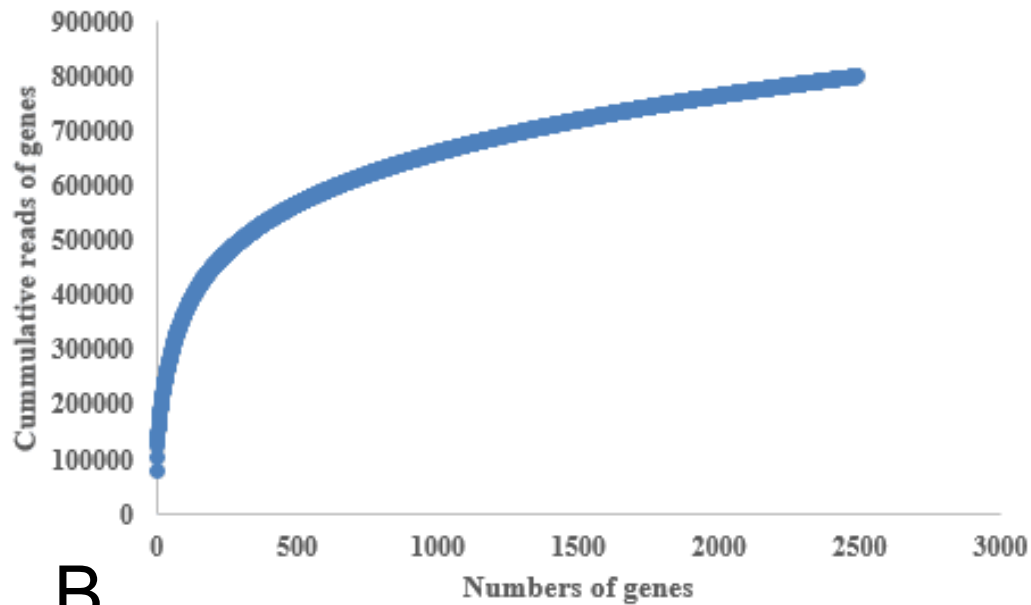

Supplement: Supplementary file 1 — The identified genes using RNA-seq in rumen under AH, CS and RS diets (A) and the predominant 2490 genes (B; covered more than 80% abundance of total mapped reads). (PDF 41 kb) [file 12864_2017_3726_MOESM1_ESM.pdf]

## COMPLEMENT AND COAGULATION CASCADES

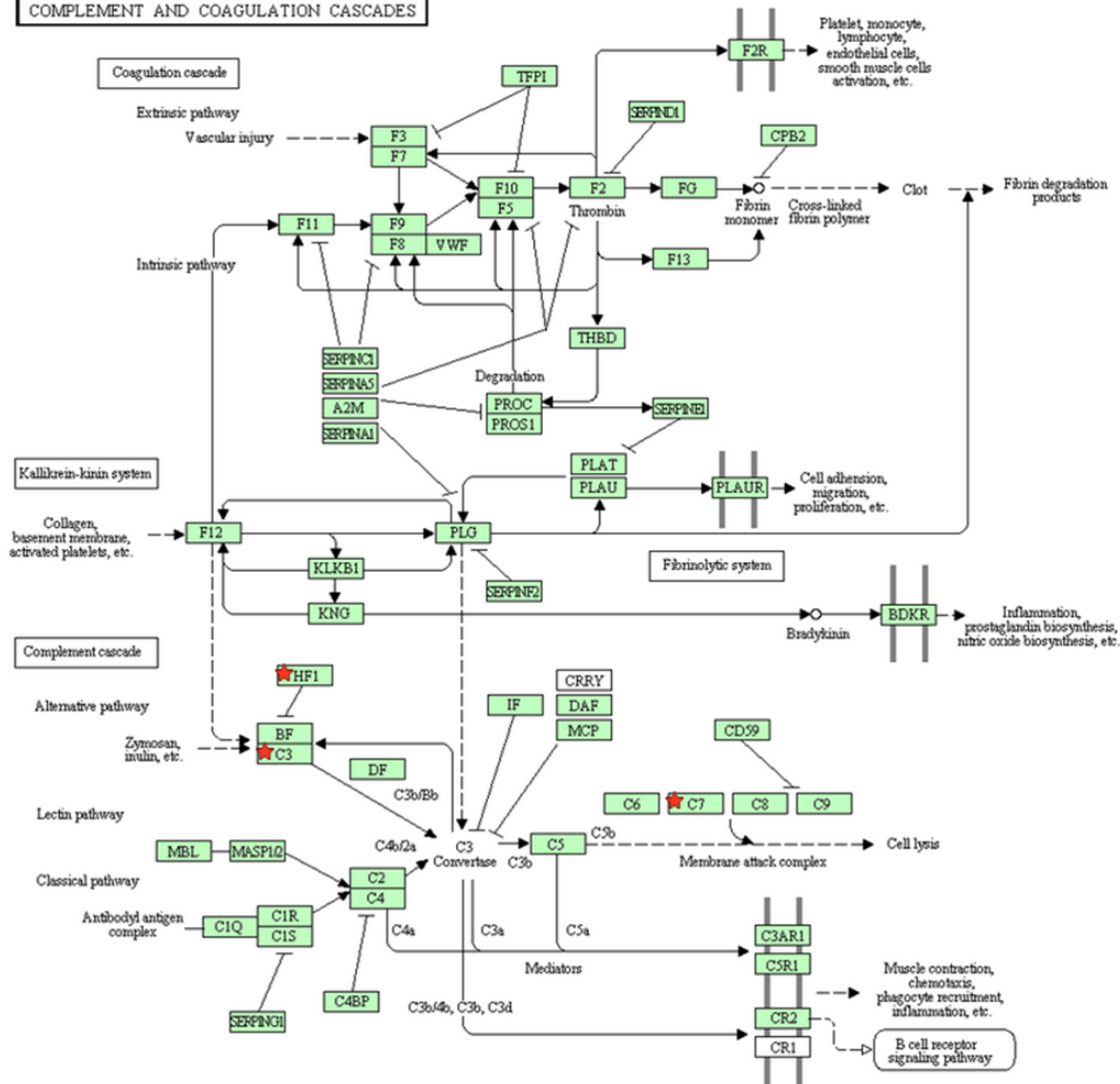

Supplement: Supplementary file 3 — The enriched pathway through the DE genes in the comparison between CS and RS. CS = Total mixed ration (TMR) containing corn stover as the main forage; RS = TMR containing rice straw as the main forage. (PDF 357 kb) [file 12864_2017_3726_MOESM3_ESM.pdf]
