# Supplementary material for: Effects of dietary physical or nutritional factors on morphology of rumen papillae and transcriptome changes in lactating dairy cows based on three different forage-based diets
Source: BMC Genomics. 2017 May 6;18:353. doi: 10.1186/s12864-017-3726-2 (PMC5420399; doi:10.1186/s12864-017-3726-2)
Supplement: Supplementary file 2 — Differentially expressed genes identified from the comparison of AH vs. CS, AH vs. RS, and RS vs. CS, respectively. (DOC 160 kb) [file 12864_2017_3726_MOESM2_ESM.doc]

Table S1 Differentially expressed genes identified from the comparison of AH vs. CS, AH vs. RS, and RS vs. CS, respectively

| Ensemble ID^1^ | Gene | log_2_FC^2^ | log_2_CPM | P-value | FDR |
| --- | --- | --- | --- | --- | --- |
| *AH vs. CS* |  |  |  |  |  |
| ENSBTAG00000043564 | *MT-ATP8* | 2.99 | 8.27 | 3.40E-34 | 2.18E-30 |
| ENSBTAG00000016209 | *SLURP1* | 1.92 | 4.81 | 4.19E-12 | 7.71E-09 |
| ENSBTAG00000002138 | *PADI1* | 1.64 | 3.73 | 4.34E-07 | 2.33E-04 |
| ENSBTAG00000016026 | *PCOLCE2* | 1.58 | 4.35 | 2.74E-06 | 1.30E-03 |
| ENSBTAG00000010991 | *TTR* | 1.57 | 3.14 | 3.57E-05 | 1.28E-02 |
| ENSBTAG00000017448 | *EFEMP1* | 1.50 | 5.45 | 3.16E-08 | 2.54E-05 |
| ENSBTAG00000002215 | *GFPT2* | 1.46 | 3.77 | 9.13E-05 | 2.80E-02 |
| ENSBTAG00000013831 | *DSG1* | 1.41 | 4.42 | 1.08E-06 | 5.57E-04 |
| ENSBTAG00000019636 | *SCARA5* | 1.29 | 4.67 | 1.63E-05 | 6.17E-03 |
| ENSBTAG00000015727 | *IFI47* | 1.25 | 4.71 | 1.25E-05 | 4.88E-03 |
| ENSBTAG00000011766 | *C7* | 1.18 | 6.35 | 1.20E-06 | 5.95E-04 |
| ENSBTAG00000012403 | *ARG1* | 1.07 | 6.60 | 5.90E-06 | 2.45E-03 |
| ENSBTAG00000047362 | *RCN1* | 1.01 | 5.14 | 1.74E-04 | 4.58E-02 |
| ENSBTAG00000016239 | *DUOXA2* | -1.18 | 5.53 | 4.71E-06 | 2.16E-03 |
| ENSBTAG00000012780 | *LPO* | -1.18 | 4.24 | 7.06E-05 | 2.22E-02 |
| ENSBTAG00000020554 | *AIF1* | -1.19 | 4.95 | 2.11E-05 | 7.74E-03 |
| ENSBTAG00000012640 | *S100A8* | -1.21 | 12.57 | 4.93E-08 | 3.73E-05 |
| ENSBTAG00000001785 | *TGM3* | -1.22 | 9.82 | 5.24E-08 | 3.74E-05 |
| ENSBTAG00000047547 | *Gm8618* | -1.24 | 6.84 | 2.10E-07 | 1.23E-04 |
| ENSBTAG00000001303 | *HSPB8* | -1.26 | 7.14 | 8.87E-08 | 5.71E-05 |
| ENSBTAG00000005012 | *HSPH1* | -1.30 | 9.03 | 7.86E-09 | 7.22E-06 |
| ENSBTAG00000001125 | *ADAM28* | -1.55 | 2.90 | 1.02E-04 | 2.97E-02 |
| ENSBTAG00000013641 | *BAG3* | -1.62 | 7.60 | 5.09E-12 | 8.19E-09 |
| ENSBTAG00000000185 | *SLC6A14* | -1.75 | 3.68 | 3.45E-07 | 1.93E-04 |
| ENSBTAG00000007402 | *ZFAND2A* | -1.81 | 4.26 | 9.95E-09 | 8.53E-06 |
| ENSBTAG00000027225 | *LAP* | -1.83 | 5.59 | 7.58E-12 | 1.08E-08 |
| ENSBTAG00000006505 | *S100A9* | -2.00 | 10.37 | 2.07E-18 | 6.67E-15 |
| ENSBTAG00000031551 | *PRSS53* | -2.33 | 5.67 | 2.44E-17 | 6.29E-14 |
| ENSBTAG00000039028 | *PI3* | -2.61 | 8.28 | 4.97E-27 | 2.13E-23 |
| ENSBTAG00000046124 | *KRT36* | -2.91 | 3.31 | 3.25E-12 | 6.96E-09 |
| ENSBTAG00000048171 | *TAP* | -3.25 | 7.95 | 8.12E-38 | 1.04E-33 |
|  |  |  |  |  |  |
| *AH vs. RS* | Gene | log_2_FC^2^ | log_2_CPM | P-value | FDR |
| ENSBTAG00000016209 | *SLURP1* | 2.45 | 4.81 | 3.63E-17 | 1.17E-13 |
| ENSBTAG00000002138 | *PADI1* | 2.30 | 3.73 | 4.01E-11 | 5.16E-08 |
| ENSBTAG00000045661 | *Metazoa_SRP* | 2.30 | 3.26 | 1.31E-06 | 6.72E-04 |
| ENSBTAG00000009844 | *CYR61* | 1.86 | 5.79 | 4.55E-13 | 9.75E-10 |
| ENSBTAG00000012918 | *CRISP3* | 1.76 | 5.82 | 4.87E-12 | 8.95E-09 |
| ENSBTAG00000008332 | *ENPEP* | 1.74 | 2.75 | 1.23E-04 | 3.17E-02 |
| ENSBTAG00000032538 | *TPRG1* | 1.71 | 3.47 | 1.96E-06 | 9.69E-04 |
| ENSBTAG00000010991 | *TTR* | 1.49 | 3.14 | 7.58E-05 | 2.03E-02 |
| ENSBTAG00000013831 | *DSG1* | 1.47 | 4.42 | 4.58E-07 | 2.56E-04 |
| ENSBTAG00000016265 | *DNAJA1* | 1.42 | 5.08 | 2.93E-07 | 1.89E-04 |
| ENSBTAG00000000522 | *AHSG* | 1.34 | 3.47 | 1.67E-04 | 3.98E-02 |
| ENSBTAG00000016357 | *VNN2* | 1.31 | 3.55 | 1.28E-04 | 3.17E-02 |
| ENSBTAG00000014069 | *PDK4* | 1.24 | 6.47 | 2.33E-07 | 1.67E-04 |
| ENSBTAG00000012403 | *ARG1* | 1.17 | 6.60 | 7.65E-07 | 4.10E-04 |
| ENSBTAG00000015241 | *EPHX2* | 1.14 | 4.59 | 4.89E-05 | 1.40E-02 |
| ENSBTAG00000002362 | *APOLD1* | 1.10 | 4.88 | 4.78E-05 | 1.40E-02 |
| ENSBTAG00000016169 | *ID1* | 1.10 | 5.82 | 1.07E-05 | 4.06E-03 |
| ENSBTAG00000002080 | *NOV* | 1.08 | 6.52 | 5.23E-06 | 2.32E-03 |
| ENSBTAG00000014774 | *ANKRD24* | 1.05 | 4.80 | 1.24E-04 | 3.17E-02 |
| ENSBTAG00000003994 | *IGFBP3* | 1.03 | 6.39 | 1.80E-05 | 6.42E-03 |
| ENSBTAG00000019870 | *SLC14A1* | 1.01 | 9.18 | 6.52E-06 | 2.70E-03 |
| ENSBTAG00000037526 | *FABP4* | 1.01 | 8.60 | 7.97E-06 | 3.21E-03 |
| ENSBTAG00000016208 | *TGM2* | -1.01 | 5.95 | 3.98E-05 | 1.22E-02 |
| ENSBTAG00000046158 | *CFB* | -1.02 | 6.90 | 1.46E-05 | 5.35E-03 |
| ENSBTAG00000001303 | *HSPB8* | -1.07 | 7.14 | 5.92E-06 | 2.54E-03 |
| ENSBTAG00000020554 | *AIF1* | -1.11 | 4.95 | 6.65E-05 | 1.82E-02 |
| ENSBTAG00000019588 | *HLA-DQB1* | -1.16 | 4.73 | 1.85E-05 | 6.43E-03 |
| ENSBTAG00000013641 | *BAG3* | -1.20 | 7.60 | 2.94E-07 | 1.89E-04 |
| ENSBTAG00000025441 | *HSPA1A* | -1.20 | 10.86 | 6.78E-08 | 5.45E-05 |
| ENSBTAG00000037605 | *HLA-DQA1* | -1.26 | 4.43 | 9.73E-06 | 3.79E-03 |
| ENSBTAG00000031160 | *IGLL1* | -1.27 | 7.05 | 4.86E-08 | 4.17E-05 |
| ENSBTAG00000001785 | *TGM3* | -1.28 | 9.82 | 1.28E-08 | 1.17E-05 |
| ENSBTAG00000012640 | *S100A8* | -1.31 | 12.57 | 4.33E-09 | 4.28E-06 |
| ENSBTAG00000010433 | *SAA1* | -1.51 | 4.53 | 3.29E-07 | 2.02E-04 |
| ENSBTAG00000007450 | *C2* | -1.53 | 4.32 | 3.45E-07 | 2.02E-04 |
| ENSBTAG00000027225 | *LAP* | -1.66 | 5.59 | 6.31E-10 | 7.38E-07 |
| ENSBTAG00000006505 | *S100A9* | -2.04 | 10.37 | 5.18E-19 | 2.22E-15 |
| ENSBTAG00000031551 | *PRSS53* | -2.16 | 5.67 | 4.83E-15 | 1.24E-11 |
| ENSBTAG00000039028 | *PI3* | -2.91 | 8.28 | 1.50E-32 | 9.66E-29 |
| ENSBTAG00000048171 | *TAP* | -3.12 | 7.95 | 3.72E-35 | 4.79E-31 |
|  |  |  |  |  |  |
| *RS vs. CS* | Gene | log_2_FC^2^ | log_2_CPM | P-value | FDR |
| ENSBTAG00000043564 | *MT-ATP8* | 2.82 | 8.27 | 5.52E-31 | 7.10E-27 |
| ENSBTAG00000002821 | *CILP* | 2.35 | 2.59 | 1.08E-05 | 6.04E-03 |
| ENSBTAG00000016026 | *PCOLCE2* | 2.31 | 4.35 | 7.20E-13 | 4.63E-09 |
| ENSBTAG00000003217 | *CADM3* | 2.04 | 2.66 | 2.30E-05 | 1.06E-02 |
| ENSBTAG00000002215 | *GFPT2* | 2.01 | 3.77 | 1.62E-08 | 2.31E-05 |
| ENSBTAG00000007312 | *Cd209f* | 1.89 | 2.83 | 2.47E-05 | 1.06E-02 |
| ENSBTAG00000019347 | *PLXDC1* | 1.83 | 3.41 | 6.55E-07 | 7.66E-04 |
| ENSBTAG00000017448 | *EFEMP1* | 1.74 | 5.45 | 1.03E-10 | 4.11E-07 |
| ENSBTAG00000019588 | *HLA-DQB1* | 1.68 | 4.73 | 2.88E-09 | 5.30E-06 |
| ENSBTAG00000019636 | *SCARA5* | 1.67 | 4.67 | 1.35E-08 | 2.18E-05 |
| ENSBTAG00000037605 | *HLA-DQA1* | 1.58 | 4.43 | 7.41E-08 | 9.53E-05 |
| ENSBTAG00000022514 | *CD5L* | 1.44 | 3.80 | 7.96E-06 | 4.66E-03 |
| ENSBTAG00000038496 | *CR2* | 1.35 | 3.83 | 6.91E-05 | 2.47E-02 |
| ENSBTAG00000009705 | *SERPINF1* | 1.34 | 4.48 | 4.26E-06 | 3.66E-03 |
| ENSBTAG00000039995 | *CFH* | 1.22 | 4.79 | 1.63E-05 | 8.42E-03 |
| ENSBTAG00000000802 | *LYVE1* | 1.22 | 4.52 | 3.26E-05 | 1.27E-02 |
| ENSBTAG00000005077 | *CXCL12* | 1.15 | 5.49 | 7.87E-06 | 4.66E-03 |
| ENSBTAG00000021176 | *CRISPLD2* | 1.15 | 6.77 | 1.12E-06 | 1.11E-03 |
| ENSBTAG00000031160 | *IGLL1* | 1.14 | 7.05 | 8.76E-07 | 9.40E-04 |
| ENSBTAG00000017280 | *C3* | 1.13 | 5.87 | 5.45E-06 | 3.89E-03 |
| ENSBTAG00000002278 | *FBN1* | 1.06 | 6.75 | 7.75E-06 | 4.66E-03 |
| ENSBTAG00000021211 | *DPT* | 1.04 | 5.77 | 2.82E-05 | 1.14E-02 |
| ENSBTAG00000012370 | *MGP* | 1.04 | 8.07 | 5.03E-06 | 3.81E-03 |
| ENSBTAG00000011766 | *C7* | 1.02 | 6.35 | 2.56E-05 | 1.06E-02 |
| ENSBTAG00000021672 | *RGS1* | -1.02 | 5.97 | 3.84E-05 | 1.45E-02 |
| ENSBTAG00000016265 | *DNAJA1* | -1.09 | 5.08 | 1.13E-04 | 3.74E-02 |
| ENSBTAG00000039991 | *UGT2B17* | -1.13 | 6.03 | 4.96E-06 | 3.81E-03 |
| ENSBTAG00000001125 | *ADAM28* | -1.87 | 2.90 | 7.82E-06 | 4.66E-03 |

^1^ AH = TMR containing alfalfa hay as the main forage; CS = TMR containing corn stover as the main forage; RS = TMR containing rice straw as the main forage.

^2^FC = fold change (AH vs. CS: AH/CS; AH vs. RS: AH/RS; RS vs. CS: RS/CS).
